# Supplementary material for: A predictive model for coupling cell division orientation to tissue mechanics during epithelial morphogenesis
Source: Front Cell Dev Biol. 2026 Jul 6;14:1844381. doi: 10.3389/fcell.2026.1844381 (PMC13381794; doi:10.3389/fcell.2026.1844381)
Supplement: Supplementary file 1 [file Supplementaryfile1.pdf]

Supplementary Materials: A Predictive Model for Coupling Cell Division Orientation to Tissue Mechanics During Epithelial Morphogenesis

1 MODEL PARAMETERS FOR DYNAMIC 3D VERTEX MODEL

As discussed in the methods section in the main text, the data reported here are from a 3D layered vertex model with active force fluctuations and overdamped dynamics. There are multiple vertex model parameters, and here we fix the values of those parameter to match data from experiments on stratified epithelia in the developing mouse embryo, using a process described in detail in that previous work Villeneuve et al. (2026). In particular, some of us previously found that there are two key parameters that differ substantially between stages E14 and E16: heterotypic interfacial tension at the apical side of basal cells σ_a and basal cell stiffness, $\Delta s = 5.4 - s_{basal}$. There is also a small difference in the wetting tension with the basement membrane, σ_b. Other vertex model parameters are identical between the two simulations, and fixed at values used previously Villeneuve et al. (2026). For completeness, we list the vertex model parameters used in simulations for this manuscript in Table S1 below:

Table S1. Simulation Parameters for E14 and E16

Parameter	E14	E16
μ	1.0	1.0
k_v	10.0	10.0
k_s	1.0	1.0
D_t	1.0	1.0
$s_{basement}$	5.14	5.14
s_{basal}	5.40	5.26
$s_{suprbasal}$	5.77	5.77
$v_{basement}$	1.0	1.0
v_{basal}	1.0	1.0
$v_{suprbasal}$	1.0	1.0
θ	$0 - \pi/2$	$0 - \pi/2$
T	0.002	0.002
T_{bot}	0.002	0.002
σ_b	0.062	0.067
σ_a	0.044	0.116
λ(divisions per basal cell per estimated day)	$0.12 - 1.95$	$0.12 - 1.95$

2 DENSIFYING PROTOCOL: CELL DIVISION WITHOUT CELL FATE SPECIFICATION

In the main text, we focused on a "homeostatic" protocol for simulations that were maintained in steady state, with constant cell number density, via cell fate specification. We made this choice to allow us to study the impact of oriented cell divisions in a tissue with fixed background properties.

However, an alternate approach is to allow cell divisions to occur without any explicit cell fate decisions/forced delaminations, and in general this will lead to a time-varying increase in the number of basal cells. We term this protocol a "densifying" protocol. As has been reported previously Fukamachi et al. (2024), such crowding is expected to eventually drive mechanical instabilities that can lead to enhanced homotypic stratifications (stratification without the need to change cell-cell interactions/mechanics), that could thereby suppress further density increases. Although previous work by some of us suggests this is not the case at later stages (E16.5) Villeneuve et al. (2026), it could be a relevant mechanism at earlier stages (E14.5).

To investigate this, we implement a model of cell division that excludes explicit cell fate specification during stratification. Here we focus on simulations with a division timescale of $t_{div} = 150$, as it is close to the rate reported in the literature for basal cells in E14-E16 (about 0.7 divisions per basal cell per day), see main text Section 3.1. We study this process for $14,400\tau$ simulation time units, which is about 24 hours and similar to an embryonic day (i.e. the amount of time between stage E14 and E15).

In this framework, the density of the basal layer is allowed to vary and stratification arises solely through homotypic interactions, without any induced changes in cell fate to facilitate layering. Here, the density of the basal layer is governed by the division angle, θ. Perpendicular divisions promote greater homotypic stratification, whereas in-plane divisions result in reduced homotypic stratification, leading to an increase in basal layer cell density. Figure S1(a,b) shows the relative change in basal cell number, $(N - N_0)/N_0$, as a function of time t for both protocols. We ran the simulations for 14400 τ (simulation time units) corresponding approximately 24 hours. Under the densification protocol (solid lines), the basal cell population increases substantially over time, whereas the constant-density protocol (dashed lines) remains close to zero throughout the simulation. The increase in basal cell number depends strongly on the division orientation angle θ, with planar divisions ($\theta = 0$) producing the largest increase $\approx 50\%$ more for E16, while perpendicular divisions ($\theta = \pi/2$) lead to a much weaker increase $\approx 30\%$. This occurs because perpendicular divisions promote stronger stratification, causing daughter cells to leave the basal layer and thereby compensating for the increase in basal cell number. Comparison between developmental stages shows that, E16 exhibits a consistently larger increase in basal cell number than E14 for all division angles. This behavior is consistent with the reduced homotypic stratification observed at E16 due to the stronger mechanical barrier between the basal and suprabasal layers, whereas the lower mechanical barrier at E14 allows more efficient stratification and delamination of basal cells.

Figure S1(c) shows the homotypic stratification fraction $N_{\text{homo}}/N_{\text{div}}$ as function of division angle for developmental stage $E14$ and $E16$, comparing homeostatic (constant density) vs. densifying protocols after 24 hours. Although at E16 the two protocols are similar for all angles, at E14 for planar and shallow divisions, the densifying protocol does generate more stratifications than the homeostatic protocol. To quantify the effect of tissue densification on stratification dynamics, we computed the difference in the stratification fraction between the densifying and constant-density protocols,

$$\Delta\left(\frac{N_{\text{strat}}}{N_{\text{div}}}\right) = \left(\frac{N_{\text{strat}}}{N_{\text{div}}}\right)_{\text{densification}} - \left(\frac{N_{\text{strat}}}{N_{\text{div}}}\right)_{\text{constant density}} \tag{S1}$$

The resulting values were then weighted by the experimentally observed distribution of division orientations, $W(\theta)$, for each developmental stage. Figure S1(d) shows that tissue densification increases the stratification fraction at E14, yielding a positive weighted difference, $\left\langle \Delta\left(\frac{N_{\text{strat}}}{N_{\text{div}}}\right)\right\rangle W(\theta) = 0.075 \pm 0.032$. In contrast, at E16 there is no difference between the two protocols (within the ensemble uncertainty),

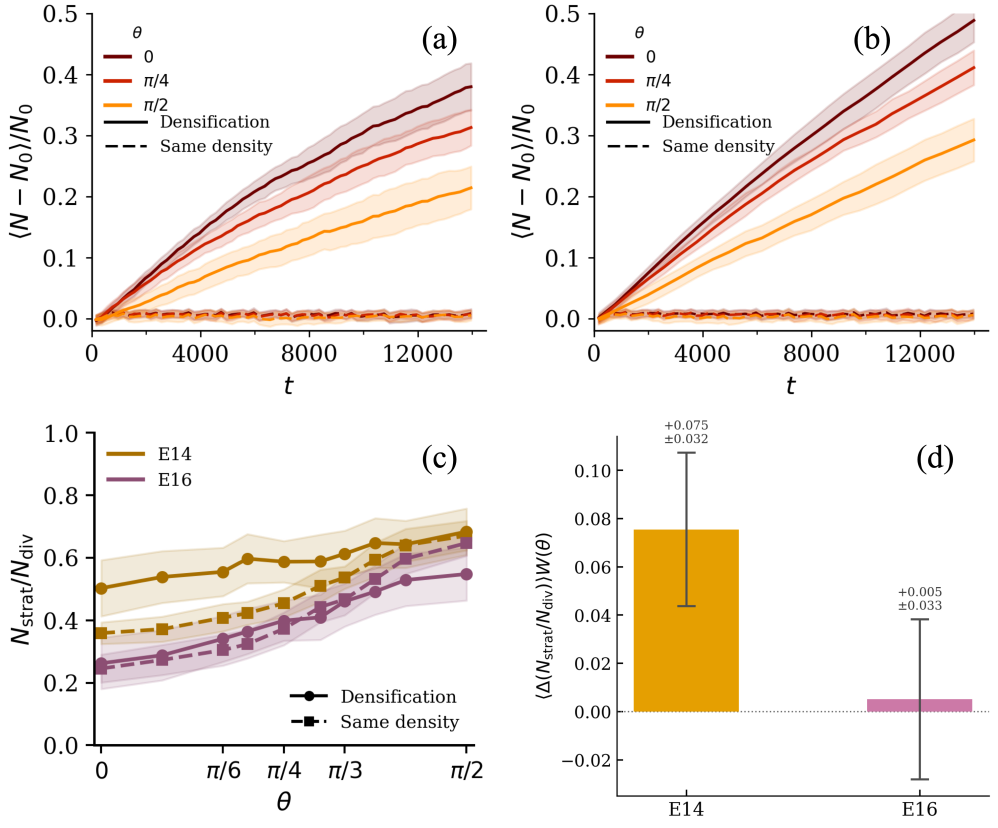

Figure S1. (a,b) Relative increase in basal cell number, $(N - N_0)/N_0$, as a function of time t (measured in units of τ) for the constant-density (dashed lines) and densifying (solid lines) protocols at developmental stages (a) E14 and (b) E16, with a division rate of $\lambda = 0.65$ divisions per basal cell per estimated day. The total simulation time corresponds to approximately 24 hours (14400τ) (c) Homotypic stratification fraction $N_{\text{homo}}/N_{\text{div}}$ as function of division angle for developmental stage $E14$ and $E16$, comparing homeostatic (constant density) vs. densifying protocols. For densifying protocols, the stratification is measured between $t = 5000 - 14,400\tau$, i.e when the densification is well underway. (d) Difference in the predicted stratification fraction – determined by weighting by the observed division planes at each stage, $\langle \Delta(N_{\text{strat}}/N_{\text{div}}) \rangle W(\theta)$, between the densifying and constant-density protocols over a 24 hour time period. In a-c, shaded regions represent the standard deviation over 40 independent simulation runs.

$\left\langle \Delta \left(\frac{N_{\text{strat}}}{N_{\text{div}}} \right) \right\rangle W(\theta) = 0.005 \pm 0.033$, indicating that densification has little measurable effect on stratification at later developmental stages. These results suggest that the mechanically softer and more permissive E14 tissue is more sensitive to crowding-induced stratification.

Thus, the two protocols should not be interpreted as mutually exclusive, but rather as limiting cases whose relative contributions shift over developmental time: early developmental stages are more permissive to density-driven stratification, whereas later stages rely increasingly on regulated, homeostatic cell-fate specification to maintain basal-layer organization and tissue turnover Villeneuve et al. (2026).

In addition, if we were to increase the rate of cell division further (i.e. $\lambda > 0.65$ divisions per basal cell per estimated day), densities would increase even further over the same time window and could eventually lead to mechanical instabilities Fukamachi et al. (2024) at both E14 and E16. This would be an interesting avenue for future research.

3 DELAY TIME FOR HOMOTYPIC STRATIFICATION

As described in the main text, homotypic stratification events were identified from cell-state time series extracted from each simulation run. A homotypic stratification event is defined as a transition in which a basal cell with identical properties to its neighbors moves upward into the suprabasal layer due to mechanical interactions. For simplicity, in the main text we focused on the total number of homotypic events that occurred at any time after a cell division, N_{homo}, but there is additional structure in how these events occur as a function of time.

For each homotypic stratification event, the delay time Δt_d was defined as the interval between the most recent time the cell entered the daughter state (after the division event) and the time at which the cell centroid breached the suprabasal layer. Two mechanistically distinct classes were distinguished: (i) immediate stratifiers, in which the cell transitioned directly, corresponding to events with $\Delta t_d \leq 10\tau$ to account for finite temporal resolution in the simulation output, and (ii) delayed stratifiers, in which the cell resided in the basal layer for a measurable duration prior to stratification, yielding $\Delta t_d > 0$.

3.0.1 Zero-Inflated Gamma Model

The distribution of delay times across all mechanical stratification events was modeled using a Zero-Inflated Gamma (ZIG) distribution. This model was chosen because the data contain a discrete point mass at zero (immediate stratifiers) superimposed on a continuous, strictly positive, right-skewed distribution (delayed stratifiers), a structure that cannot be captured by a standard unimodal distribution. The ZIG model is defined as a two-component mixture Lee and Haran (2025):

$$P(\Delta t) = p_0\delta(\Delta t = 0) + (1 - p_0)\Gamma(\Delta t|k, m), \Delta t > 0 \tag{S2}$$

where $p_0 \in [0, 1]$ is the weight representing the probability of immediate stratification, δ is the delta function, and $\Gamma(k, m)$ is the two parameter Gamma distribution with shape parameter $k > 0$ and scale parameter $m > 0$. The mean and variance of the non-zero component are $\mu_{nz} = km$ and $\sigma^2_{nz} = km^2$ respectively.

Figure S2 shows the dependence of the fitted ZIG parameters on the division angle for two developmental stages, $E14$ and $E16$. Figure S2 (A) shows the zero-inflation parameter p_0, which represents the probability of immediate stratification. In both developmental stages, p_0 varies systematically with θ, indicating that the likelihood of immediate stratification depends on the angle of division. For symmetric divisions ($\theta \approx 0$), the cleavage plane produces two daughter cells that are mechanically and geometrically equivalent with respect to their surrounding environment. This symmetry suppresses any immediate directional bias or force imbalance that could drive stratification at the moment of division. As a result, stratification usually does not occur instantaneously, leading to a reduced probability of immediate stratification (lower p_0) and longer delay times. In this regime, additional time is required for stochastic fluctuations, local rearrangements, or external mechanical cues to break the initial symmetry before stratification can proceed. In contrast, highly oblique divisions ($\theta \approx \pi/2$) inherently introduce strong geometric and mechanical asymmetries between daughter cells. This built-in asymmetry increases the likelihood of immediate stratification, reflected by a higher p_0, and reduces the characteristic delay time for the remaining delayed events. Intermediate division angles interpolate between these two limits, generating a mixture of immediate and delayed stratification events. The smooth angular dependence of both p_0 and the mean nonzero delay time indicates that the division angle acts as a continuous control parameter that regulates how efficiently mechanical asymmetry is generated at division.

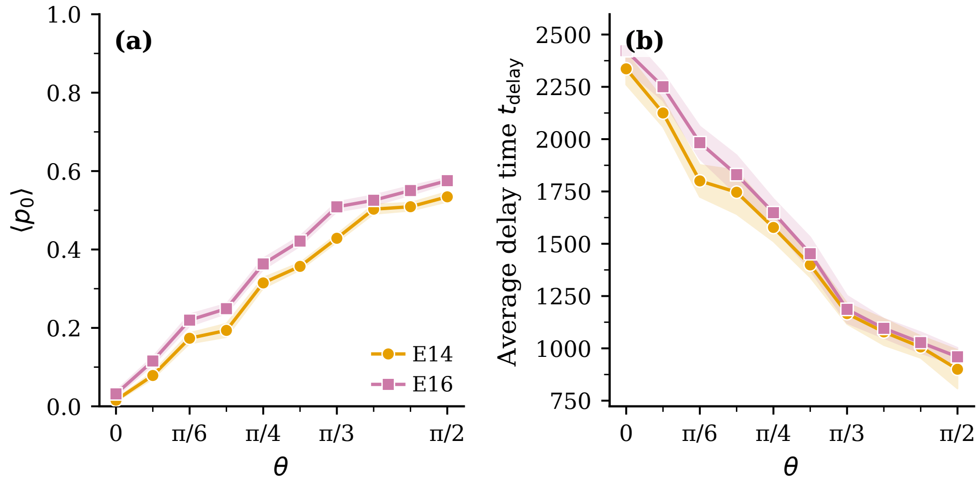

Figure S2. Dependence of stratification delay statistics on the division angle θ at a fixed division rate. (a) Zero–inflation parameter p_0, representing the probability of immediate stratification ($\Delta t \leq 10\tau$), as a function of the division angle θ for developmental stages E14 (orange) and E16 (pink) at $\lambda = 0.49$ divisions per basal cell per estimated day. (b) Mean delay time of the nonzero component of the distribution as a function of θ for the same developmental stages and rate. Shaded regions denote the standard error of the mean (SEM). Only selected values of θ are labeled on the horizontal axis for clarity.

Figure S2 (B) shows the mean delay time of the nonzero component of the distribution, μ_{nz}, as a function of θ. This quantity characterizes the typical waiting time for delayed stratification events. For symmetric divisions ($\theta \approx 0$), the two daughter cells are created in a mechanically equivalent configuration. In the absence of an intrinsic asymmetry at division, stratification cannot proceed immediately and instead requires a subsequent symmetry-breaking process. This may arise from stochastic fluctuations, gradual force redistribution, or interactions with neighboring cells. As a consequence, stratification occurs only after a prolonged waiting period, leading to larger mean delay times for symmetric divisions. In contrast, highly oblique divisions ($\theta \approx \pi/2$) generate a pronounced mechanical asymmetry at the moment of division. Differences in geometry, force transmission, or coupling to the surrounding tissue provide an immediate directional bias that facilitates stratification. When stratification is delayed in this regime, the required mechanical reorganization is minimal, resulting in significantly shorter delay times compared to symmetric divisions. Intermediate angles exhibit delay times that interpolate between these two limits, consistent with a gradual increase in division-induced asymmetry as θ increases.

4 DIVISION-INDUCED BASAL-LAYER FLUIDIZATION

We compute the squared separation between daughter cells (Equation 6 in the main text) within the basal layer as a function of time, division orientation, and division rate, and use this to estimate effective diffusion (rearrangement) rates. Figure S3(a) shows the diffusion coefficient extracted from the MSD for different division rates as a function of the division angle θ. The diffusion depends strongly on division rate, with faster divisions leading to increased rearrangements. In contrast, the dependence on division orientation is weak and remains nearly constant.

The orange bars in Fig S3(b) show the diffusion data for E14 from Fig S3(a), averaged over orientation angle. Specifically, the average is weighted by the probability of observing a given orientation at E14 (see main text Eq. 13 and Fig 2(c).) The pink bars show the same averaging for E16 simulation data. As already

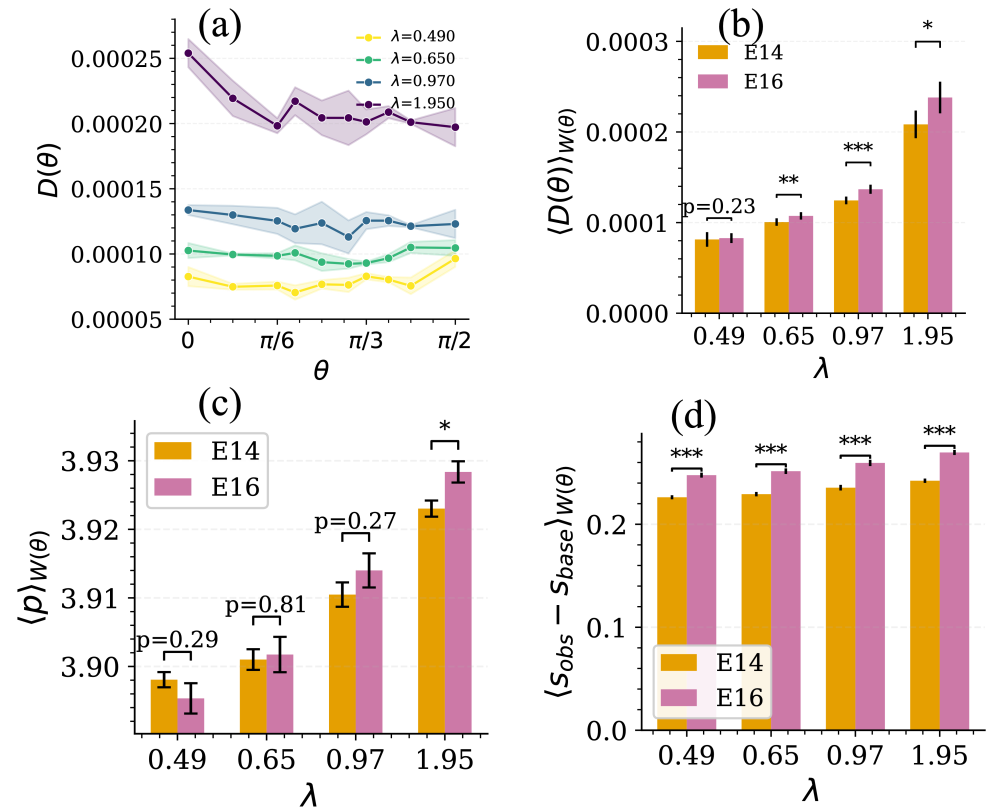

Figure S3. (a) Diffusion coefficient from daughter-cell separation as a function of division rate and angle θ at $E14$. (b–d) Probability-weighted averages (using experimental orientation distributions) of diffusion coefficient (b), 2D shape index (c), and change in observed 3D shape index or stiffness (d) at $E14$ and $E16$ across division rates. Error bars denote standard deviation over orientations.

discussed in the main text for E14 data, the diffusion is larger when there is faster rates of division (i.e. $\lambda = 1.95$ divisions per basal cell per estimated day). The E16 data shows the same overall trends, and for the faster division rates E16 is slightly more diffusive than E14.

We next examine whether cell shape correlates with basal-layer rearrangement dynamics. To this end, we quantify changes in basal-layer stiffness via $\Delta s = s_{\rm obs} - s_{\rm base}$, where $s_{\rm obs}$ is the 3D shape of basal cells in the presence of divisions and $s_{\rm base}$ is the corresponding shape in the absence of divisions. The 3D shape index is defined as $s = A/V^{2/3}$, where A and V are the cell surface area and volume, respectively. The 2D shape index is defined as $p = P/\sqrt{A_p}$, where A_p and P are the area and perimeter of the mid-plane cross-section of a basal cell.

Figure S3(c,d) compares 2D and 3D shape indices at developmental stages $E14$ and $E16$, again weighted by then probability of an orientation being experimentally observed at each stage, across division rates. Both metrics show increased fluidization with increasing division rate, as reflected by higher shape indices, although the 2D metric is much more strongly correlated with diffusion than the 3D metric. This trend is consistent across stages, with a slight enhancement of fluidization and higher shape index at $E16$ compared to $E14$.

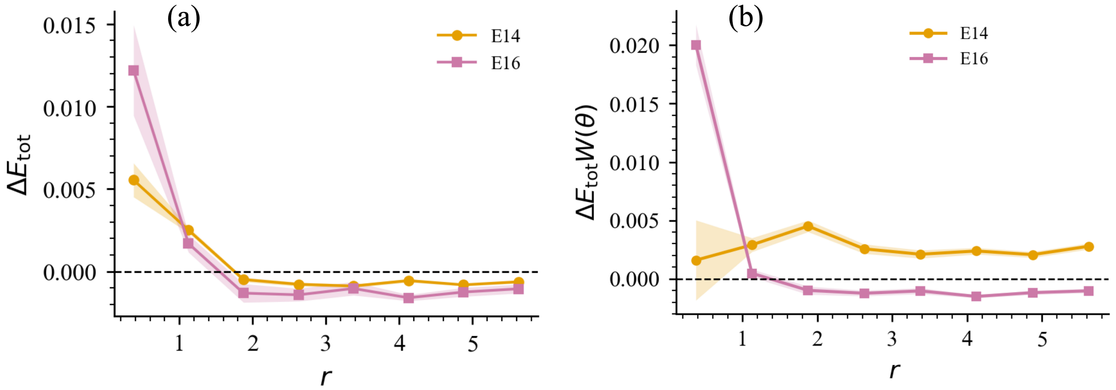

Figure S4. Mechanical energy perturbation induced by cell division in neighboring basal cells as a function of radial distance r from the dividing cell. Energy differences were calculated between the onset of cell growth and immediately after division. (a) Radial profile of the total energy difference (b) Probability-weighted average change at division rate $\lambda = 0.65$. Shaded bands denote the SEM calculated from ensemble averages over 40 independent realizations.

5 ENERGY INJECTED DUE TO DIVISION

To assess how cell division perturbs the mechanical energy of surrounding tissue, we computed the energy of neighboring basal cells as a function of radial distance from the dividing cell, comparing two time points – when the cell starts to grow ($t_{initial}$) and immediately after the division event (t_{after}). The total energy is calculated using Eq. 1 (main text). The difference in energy is given by $\Delta E_{tot} = E_{after} - E_{initial}$.

Figure S4 (a) shows the change in total energy ΔE_{tot} as a function of radial distance from the dividing cell for embryonic stages E14 and E16. In both stages, cells closest to the dividing cell $r < 1$ experience the largest increase in energy, indicating that the division event generates a strong local mechanical perturbation. However, E16 exhibits substantially larger energy injection at short range than E14, suggesting that the stiffer late-stage tissue confines more of the division-induced mechanical fluctuations within the basal layer rather than dissipating them into surrounding tissue layers. As the distance from the division site increases, the energy difference rapidly decreases and approaches zero around $r \approx 1.5$. Interestingly, this length scale is comparable to the characteristic daughter–daughter displacement distance measured in our simulations, $\sqrt{2.5} \approx 1.58$, corresponding to approximately one to two typical cell diameters. This suggests that the enhanced mechanical fluctuations in E16 remain spatially localized near the division site and do not propagate efficiently beyond the immediate cellular neighborhood. To account for the experimentally measured distribution of division angles at each developmental stage, we also weight the energy distribution observed for different division orientations by the probability $W(\theta)$ of observing each division orientation (Figure S4(b)). This demonstrates that in the experimental conditions with varied orientation planes, divisions at E16 are likely injecting more localized mechanical stress around the division site as compared to E14.

6 DESCRIPTION OF SUPPLEMENTARY MOVIES

- M1: Homotypic stratification of basal cells at developmental stage $E14$ and division rate of $\lambda = 0.65$ divisions per basal cell per estimated day. Orange-colored cells represent cells that have undergone homotypic stratification, acquiring a differentiated suprabasal type.

- M2: Stratification based on basal cell fate specification at developmental stage $E14$ and $\lambda = 0.65$ divisions per basal cell per estimated day. Magenta-colored cells indicate cells that are mechanically induced to stratify in order to maintain a constant basal layer density.
- M3: Dynamics of symmetric division ($\theta = 0$) at developmental stage $E14$ with $\lambda = 0.65$ divisions per basal cell per estimated day.
- M4: Dynamics of asymmetric division ($\theta = \pi/2$) at developmental stage $E14$ with $\lambda = 0.65$ divisions per basal cell per estimated day

REFERENCES

Fukamachi, S., Datta, R., Wuergezhen, D., Ichikawa, T., Yagasaki, R., Leeaw, P., et al. (2024). Epithelium stratifies via nucleation and growth induced by foam-geometric instability. *bioRxiv* , 2024–12

Lee, B. S. and Haran, M. (2025). A class of models for large zero-inflated spatial data: Bs lee, m. haran. *Journal of Agricultural, Biological and Environmental Statistics* 30, 746–768

Villeneuve, C., Hassikpezi, S. A. E., Albu, M., Ruebsam, M., Biggs, L. C., Vinzens, S., et al. (2026). Tissue-scale mechanics controls differentiation strategy and dynamics of epithelial multilayering. *bioRxiv* , 2026–02
